# Solitary Fibrous Tumors of the Chest: An Analysis of Fifty Patients

**DOI:** 10.3389/fonc.2021.697156

**Published:** 2021-07-01

**Authors:** Jingwen Zhang, Jumin Liu, Zhihao Zhang, Beizong Tian

**Affiliations:** ^1^Department of Geriatric Respiratory and Sleep, Henan Institute of Respiratory Diseases, The First Affiliated Hospital of Zhengzhou University, Zhengzhou, China; ^2^Department of Infectious Diseases, The First Affiliated Hospital of Zhengzhou University, Zhengzhou, China

**Keywords:** solitary fibrous tumor of the chest, characteristic, diagnosis, treatment, retrospective analysis

## Abstract

**Background:**

A solitary fibrous tumor of the chest (SFTC) is a subtype of solitary fibrous tumor (SFT) with a low incidence rate. The purpose of this study is to analyze the diagnosis and treatment of SFTC and the difference between benign and malignant solitary fibrous tumor of the pleura (SFTP) to improve the understanding of this rare disease.

**Methods:**

A retrospective analysis of fifty patients with SFTC (33 cases in the pleura and 17 in the lung) was performed. Clinical and imaging characteristics, pathological features, and treatment follow-up outcomes were analyzed.

**Results:**

The common symptoms of the 50 patients included a cough, expectoration, chest tightness, fever, and chest pain. Space occupying lesions were found *via* plain computed tomography (CT) and enhanced CT was used for enhancement of the tumors. It was also found that 18 cases had necrosis, and 5 cases had calcification. The histopathology results showed that frequent nuclear division, obvious morphological variation, necrosis, and the high expression of Ki-67 cells are markers of malignant SFTC. There were significant differences in age, chest tightness, necrotic foci in CT, and expression of Ki-67 between the benign and malignant SFTP cases. All the patients who received treatment were given an excellent prognosis.

**Conclusion:**

A combination of enhanced CT, histopathology, and immunohistochemistry can be used for the accurate diagnosis of SFTC. Advanced age, chest tightness, necrotic foci in CT, and a high Ki-67 index were more likely to be malignant SFTP. Operation and radiofrequency ablation can provide favorable outcomes for both benign and malignant SFTC.

## Introduction

Solitary fibrous tumors (SFTs) are a type of mesenchymal tumor. These tumors originate from CD34^+^ dendritic mesenchymal (stem) cells and possess the potential to form into fibroblasts, myofibroblasts, or vascular endothelial cells (1). The site of SFTs is extensive and includes the pleurae, head and neck, extremities, meninges, lungs, liver, kidneys, pancreas, prostate, thymus, and bones (2–7). SFTs of the chest (SFTC), which mainly consist of SFTs of the pleura (SFTP) and the lung (SFTL), are a subtype of SFT. SFTP account for 5% of all pleural tumors, ranking second after mesothelioma. Malignant solitary fibrous tumors (MSFTs), which are extremely rare, account for 10–15% of SFTP (1). Accurate diagnosis and effective treatment for SFTC are obviously beneficial to this type of patient. Therefore, it is vital to systematically analyze the diagnosis and treatment of SFTC to provide evidence for clinicians.

Chest computed tomography (CT), histopathology, and immunohistochemistry are the current methods used for diagnosis. However, there is still no uniform CT and histopathology phenomenon standard for distinguishing benign and malignant SFTC. For example, Cardillo et al. (8) identified risk factors for the malignancy of SFTC as being the uneven density of the tumor, an unclear boundary, a thickened surrounding pleura, and excessive length (>10 cm). On the other hand, England et al. (9) found that rich and dense tumor cells, moderate to severe pleomorphism, necrosis, and pathological mitosis always indicate malignancy of SFTC. In addition, few studies have revealed the outcomes of SFTC after treatment. Therefore, analyzing diagnostic methods and prognosis after treatment is vital.

In this study, we analyzed the clinical features, including the symptoms, signs, imaging, and pathology, of 50 cases of SFTC. Then, we explored the treatment and outcomes of all the patients, and finally, we compared the difference between benign and malignant SFTP.

## Methods

### Participants

Fifty patients with SFTC treated at the Department of Thoracic Surgery of The First Affiliated Hospital of Zhengzhou University between January 2012 and June 2020 were enrolled in this research. The location of these SFTC were in the pleura (n=33) and the lung (n=17). All SFTC were initially diagnosed *via* CT and confirmed through histopathology. All tumors were risk-stratified by using the modified scheme proposed by Demicco et al. (10). This study was approved by the First Affiliated Hospital of Zhengzhou University and all participants signed informed consent.

### Diagnosis

A diagnosis of SFTC depends on CT, histopathology, and immunohistochemistry. All the patients here underwent a plain CT scan, and 48 patients received an enhanced CT scan, and histopathology and immunohistochemistry were performed on all samples. For immunohistochemistry, the presence and level of anti-cluster of differentiation (CD)34 antibodies, vimentin, Bcl-2, signal transducer and activator of transcription 6 (STAT6), CD99, epithelial membrane antigen (EMA), smooth muscle actin (SMA), S-100, cytokeratin (CK), desmin, and Ki-67 were determined.

### Treatment

Among the 33 patients with SFTP, 27 patients selected surgery as the initial treatment, 18 of them choosing open surgery and 9 of them opting for thoracoscope surgery. Two patients chose interventional treatment for their initial treatment. One of these 2 patients underwent CT-guided radiofrequency ablation of a mediastinal solitary fibroma as the initial treatment in the first month, the third month, and the seventh month after the diagnosis was confirmed. The other case underwent right diaphragmatic artery angiography and embolization, followed by radiofrequency ablation in the first month, second month, and sixth month, and thoracoscopic tumor resection in the fourteenth month. After surgery, 25 patients were selected for dynamic observation and follow-up, and one patient with a benign SFTP received deep hyperthermia (4 times) over a 4-month period, and one patient received docetaxel and carboplatin chemotherapy (4 times). The remaining four SFTP patients failed to undergo any treatment.

All the 17 SFTL patients chose surgery, 7 of them selecting open surgery and 10 of them thoracoscopic surgery. None of them received adjuvant treatment after surgery.

### Follow-Up

Death of any cause was selected as the primary end point, and recurrence of tumor was identified as the secondary end point. Nine cases were lost to follow-up, and the follow-up period for the other patients was 3–99 months, the last follow-up being on 25 September 2020. Telephone and outpatient follow-up were used, and the patients received routine follow-up, including chest CT and clinical examinations, every six months after treatment.

### Statistical Analysis

SPSS 22.0 was used for statistical analysis. The measurement data with normal distribution were expressed by xˉ ± s, and the measurement data that did not conform to the normal distribution were represented by median (M) (25th percentile, 75th percentile) (Q1, Q3). A Student’s t-test or rank sum test was employed for the comparison of measurement data between two groups, and the exact probability method was used to compare the two groups of classified data. A p-value < 0.05 indicated statistical significance.

## Results

### Baseline Characteristics of Patients

The baseline characteristics of the patients are listed in **Table 1** and **Supplement Table 1**. Among the 33 SFTP patients, there were 19 cases with a tumor in the left pleura and 14 cases with one in the right pleura, 25 of the tumors being benign and 8 malignant. Besides that, only 2 patients had metastases (1 with liver metastasis, and 1 with multiple lung metastases). All the 17 SFTL cases were benign tumors, with 8 in the left lung and 9 in the right lung. The ages of the 50 patients ranged from 28 to 84, the median age being 53 years. Among the 50 patients, 21 were male and 29 were female. None of the patients had a history of asbestos exposure but one male patient had worked in a coal mine. Only 10 of the 50 patients had a history of heavy smoking (smoking index more than 300). In addition, we compared the baseline feature of benign and malignant SFTP patients. The gender, location, and hospital time were similar in the two groups (all p > 0.05), but the mean age in the malignant group was higher than that in the benign group (p = 0.049) (**Table 2**).

**Table 1:** Baseline characteristics of patients.

Characteristics		SFTP^1^	SFTL^2^
Age	≤55	20	11
	>55	13	6
Gender	Male	13	8
	Female	20	9
	Benign	25	17
	Malignant	8	0
Smoking	Heavy smoking	6	4
	Light / No smoking	27	13
Location	Left mediastinum	6	
	Left chest cavity	13	
	Right mediastinum	2	
	Right chest cavity	12	
	Left lung		8
	Right lung		9
Symptom	Cough	12	7
	Expectoration	8	5
	Chest tightness	9	2
	Fever	0	1
	Chest pain	6	4

^1^SFTP, solitary fibrous tumors in pleura.

^2^SFTC, solitary fibrous tumors in lung.

**Table 2:** Comparison between benign and malignant SFTP patients.

Characteristics		Benign	Malignant	P
Age		51.0 (42.6,57.0)	59.5(44.0,60.8)	0.049
Gender	Male	9	4	0.681
	Female	18	4	
Hospital time		18.00 ± 4.70	22.13 ± 10.62	0.318
Smoking	Heavy smoking	5	1	1
	Light / No smoking	20	7	
Location	Left mediastinum	3	3	0.416
	Left chest cavity	10	3	
	Right mediastinum	2	0	
	Right chest cavity	10	2	
Symptom	Cough	8	4	0.42
	Expectoration	6	2	1
	Chest tightness	4	5	0.02
	Fever	0	0	1
	Chest pain	5	1	1
Longest diameter	<5cm	2	2	0.968
	5-10cm	7	1	
	>10cm	16	5	
Margin	clear	23	6	0.241
	fuzzy	2	2	
Density	uniform	4	2	0.616
	uneven	21	6	
Calcification	Yes	3	0	0.56
	No	22	8	
Necrosis	Yes	10	7	0.039
	No	15	1	
Enhancement	mild	9	4	1
	mild to moderate	4	0	
	moderate	7	2	
	obvious	5	2	
Intratumoral blood vessel	Yes	8	4	0.42
	No	17	4	

The results of the modified Demicco risk stratification model showed that among the patients with SFTP, there were 22 with low risk, 11 with medium risk; among the patients with SFTL, 16 patients with low risk and 1 patient with medium risk. There were 19 benign MSFT patients with low risk, 6 moderate risk; 3 malignant MSFT patients with low risk, 5 with moderate risk. There was no significant difference between benign and malignant MSFT groups (*P*=0.082) (see details in **Table 3**).

**Table 3:** Risk stratification systems for solitary fibrous tumor proposed by Demicco (Modified).

Criterion	Score
Age, years	
<55	0
≥55	1
Size, cm	
<5	0
5 to <10	1
10 to <15	2
≥15	3
Mitoses/10 hpf	
0	0
1-3	1
>4	2
Necrosis	
Absent	0
Present	1
Risk	Total
Low	0-3
Moderate	4–5
High	6–7

The main symptoms of the 50 patients were a cough, sputum production, chest tightness, and chest pain, while the main signs observed were dull or solid percussion sounds on the side of the chest where the tumor lay and reduced or diminished breath sounds during auscultation. In addition, we compared the differences between the signs and symptoms of the benign and malignant SFTP patients. The incidence of cough, sputum production, and chest pain in the two groups was similar (all p > 0.05), while patients in the malignant group exhibited a higher incidence rate of chest tightness (p = 0.02).

### The Diagnosis for SFTC

**Figure 1:**
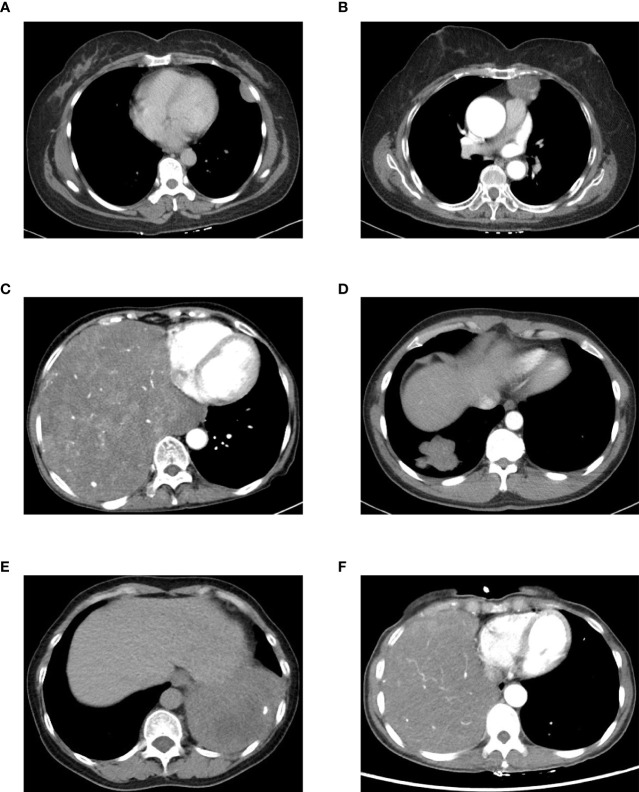
CT features of the 50 SFTC patients: **(A)** a mound-like lesion with uniform internal density in the left pleura; **(B)** a circular lesion in the left mediastinum with vascular shadows inside; **(C)** a huge lesion in the right thoracic cavity with uneven internal density, showing uneven enhancement; **(D)** a lobular lesion in the right lung with burr signs; **(E)** a lesion with calcification and necrosis in the left thoracic cavity; and **(F)** a lesion with multiple tortuous vascular shadows in the right thoracic cavity.

**Figure 2:**
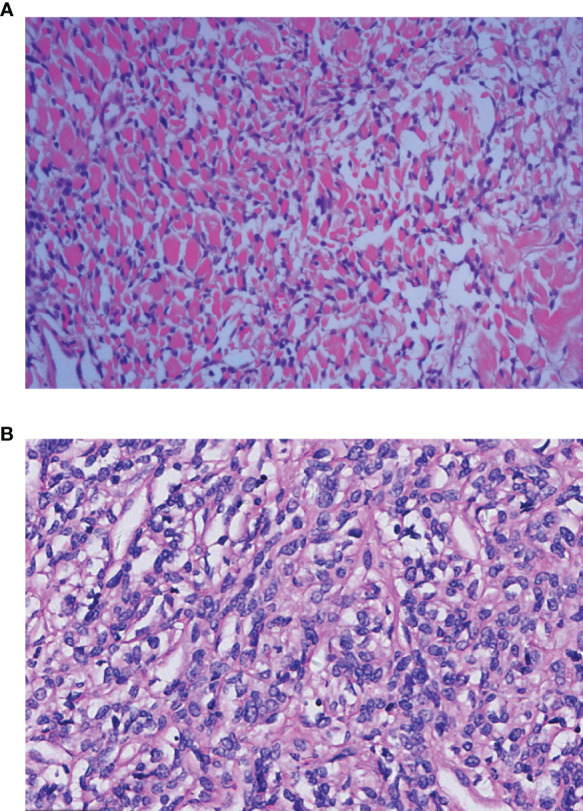
Histopathological features of the benign and malignant SFTP patients: **(A)** The benign cells are spindle-shaped or short spindle-shaped and grow randomly without structure [Hematoxylin Eosin(HE) staining×10]; and **(B)** the malignant cells are abundant and dense, and obvious atypia and mitoses can be seen (HE×10).

The diagnosis of SFTC depends on CT, histopathology, and immunohistochemistry. All the patients underwent a preoperative plain CT scan, and 48 of them also underwent a preoperative enhanced CT scan (**Table 4**). The characteristics of the plain CT were listed as follows: (1) the tumors were mound-shaped (3 cases) (**Figure 1A**), round, or oval (34 cases) (**Figure 1B**), with a growth along the thoracic cavity (13 cases) (**Figure 1C**), or lobes and burrs (1 cases) (**Figure 1D**); (2) the tumor size ranged from 1.8 to 21.5 cm, and the average size was 10.56 ± 3.18cm; (3) the density of 18 cases was uniform, and that of 32 cases was uneven; and (4) 5 cases had calcification, and 18 cases had necrosis (**Figure 1E**). Uneven enhancement was seen in all the samples undergoing enhanced CT, and 12 cases had mild enhancement, 8 cases had mild to moderate enhancement, 16 cases had moderate enhancement, and 12 cases had strong enhancement. In addition, 16 of the enhanced CT cases contained multiple tortuous vascular shadows (**Figure 1F**). By comparing the CT results of the benign and malignant SFTC, we found that patients in the malignant group showed higher necrosis (p = 0.039). However, there was no statistical difference in the size, density, boundary situation, intratumoral blood vessels, and the degree of enhancement of the lesions (all p > 0.05) (**Table 2**).

**Table 4:** CT features of the 50 patients.

Characteristics		Number of patients
Shape	mound-shaped	3
	round or oval	34
	along the thoracic cavity	13
Longest diameter	<5cm	11
	5-10cm	14
	>10cm	25
Margin	clear	43
	fuzzy	7
Density	uniform	18
	uneven	32
Calcification		5
Necrosis		18
Enhancement	mild	12
	mild to moderate	8
	moderate	16
	obvious	12
Intratumoral blood vessel	Yes	16
	No	32

All 50 patients underwent a CT-guided lung biopsy before treatment, and subsequently immunohistochemistry was carried out on the specimens. Forty-eight cases were confirmed as being solitary fibrous tumors, but two patients lacked a clear diagnosis and this was finally confirmed by pathology after surgery. The characteristics of benign SFTC were as follows: (1) the tumor cells were oval or short fusiform in shape and distributed unevenly in the matrix; (2) tumor cells mainly exhibited an unstructured random growth pattern; (3) the abnormality of the tumor cells was not significant; and (4) the nucleus was round or oval, and 0–4 mitotic were seen per high power field (HPF) (**Figure 2A**), while malignant tumor cells mainly showed nuclear pleomorphism and hypermitosis, accompanied by infiltrating borders and tumor necrosis (**Figure 2B**).

**Figure 3:**
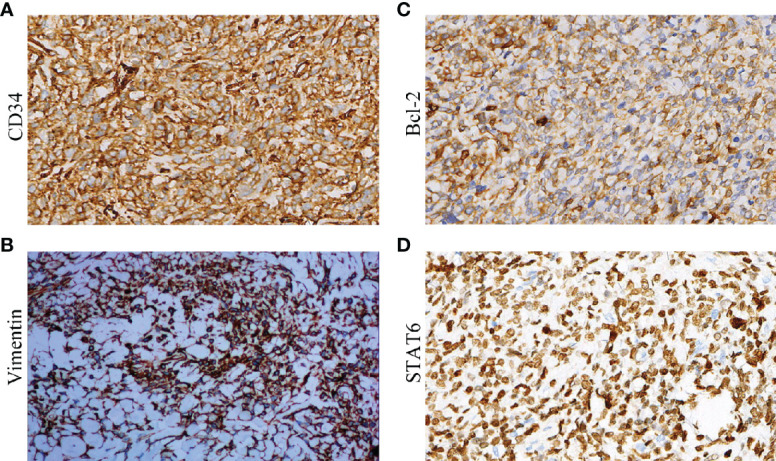
Immunohistochemistry of biological markers for SFTP: **(A)** CD34; **(B)** Vimentin; **(C)** Bcl-2; and **(D)** STAT6.

Immunohistochemistry was performed on all the 50 specimens, and the presence and levels of CD34, Vimentin, Bcl-2, STAT6, CD99, EMA, SMA, S-100, CK, Desmin, and Ki-67 were detected. The positive rates for CD34, Vimentin, Bcl-2, STAT6, CD99, EMA, SMA, S-100, CK, and Desmin were 100%, 100%, 98%, 96%, 90%; 12%, 10%, 0%, 0%, and 0%, respectively (**Figure 3**). The Ki-67 results showed that the proliferation index of benign SFTC is 1–10% and that of malignant SFTC is 5–70%. An analysis showed that there were no differences in CD34, Vimentin, Bcl-2, STAT6, CD99, EMA, SMA, S-100, CK, and Desmin between benign and malignant SFTP patients, but the Ki-67 index of malignant SFTP was significantly higher than that of benign SFTP (**Table 5**). These findings highlight the importance of CD34, Vimentin, Bcl-2, STAT6, and CD99 in the diagnosis of SFTC, and of Ki-67 in the differentiation between benign and malignant SFTC.

**Table 5:** Pathological immunohistochemical characteristics of benign and malignant SFTP patients.

Pathological immunohistochemical characteristics		Benign	Malignant	P
CK	Positive	0	0	1.000
	Negative	25	8	
Vimentin	Positive	25	8	1.000
	Negative	0	0	
CD34	Positive	25	8	1.000
	Negative	0	0	
S-100	Positive	0	0	1.000
	Negative	25	8	
bcl-2	Positive	25	8	1.000
	Negative	0	0	
EMA	Positive	3	2	0.574
	Negative	22	6	
SMA	Positive	2	2	0.241
	Negative	23	6	
CD99	Positive	23	6	0.241
	Negative	2	0	
Desmin	Positive	0	0	1.000
	Negative	25	8	
STAT-6	Positive	25	8	1.000
	Negative	0	0	
Ki-67	>5%	5	8	0.0001
	≤5%	20	0	

Overall, CT, histopathology, and immunohistochemistry can be used for the diagnosis of SFT and for the identification of benign and malignant SFTC. Nuclear pleomorphism and hypermitotism, accompanied by infiltrating borders, tumor necrosis, and a high Ki-67 index can be used as pathological markers of malignant SFTC.

### Follow-Up

Only two patients died. One patient with benign SFTC in the left pleura died of sudden acute myocardial infarction 2 months after the diagnosis (without treatment), and one patient with malignant SFTC in the left pleura died 5 months after the diagnosis because of respiratory failure (without treatment). Both of the patients were more than 80 years old.

Only one patient experienced a recurrence of the tumor. This patient, who had malignant SFTC in the left pleura, relapsed 79 months after surgery. He was given further surgery and adjuvant chemotherapy, and a favorable prognosis was obtained.

In general, the prognosis for patients undergoing either open or thoracoscopic surgery was excellent.

## Discussion

Previous studies showed that malignant SFTP, a rare tumor of the chest, accounted for 10–15% of the total number of SFTP (1), but in this study the figure was 21.2%, suggesting that the incidence of malignant SFTP may be increasing. People tend to develop SFTs between 50 and 70 years of age (11). The median age of the patients in this study was 53 years old, but the range of age was large, with the incidence of SFTs occurring in all ages from 28 to 84 years, suggesting that the disease can occur in all ages, and clinicians should bear this in mind. In this study, physical examination of 17 of the 50 patients (34%) showed them to have the disease, indicating that the onset of the disease is insidious. However, symptoms such as pain, coughing, and difficulty in breathing often occur in the middle and late stages (12, 13). Therefore, appropriate and accurate diagnostic methods are needed. In plain CT scans, the main manifestations of SFTC are single space-occupying lesions in the thoracic cavity or in the lung, with different tumor sizes, smooth borders, and occasionally shallow lobes. The density varies based on the size of the tumor. In detail, smaller tumors show more uniform density while larger ones show less uniform density. In enhanced CT scans, smaller tumors are enhanced more evenly, while larger tumors exhibit clear uneven map-like enhancement. Multiple blood vessels can also be seen in the mass in the arterial phase, and there is obvious enhancement of the parenchymal part of the tumor in the venous phase, while delayed scanning is still enhanced. In this study, we found plain and enhanced CT manifestations like those previously reported. Earlier studies have also concluded that preoperative lung histopathology is not meaningful for the diagnosis of SFTC. However, in this study 96% of SFTC cases could be clearly diagnosed by CT-guided lung histopathology before surgery. Hence, we would still recommend carrying out histopathology for patients suspected of having this disease. SFTC originates from CD34^+^ dendritic mesenchymal (stem) cells. Hence, CD34 is usually considered to be a diagnostic marker for SFTC (14), and it has been found that the positive rate of CD34 in SFTC is about 79–100% (15, 16). Indeed, all of the patients in our study were CD34^+^. A previous study found that Bcl-2 possesses a higher positive rate compared to CD34 (17), but we found that the positive rate of Bcl-2 was 98%. There have been reports of a highly sensitive and specific NGFI-A–binding protein 2–STAT6 fusion gene in benign or malignant SFTC. The detection of STAT6 protein by immunohistochemistry can indirectly reflect information about the fusion gene, thereby replacing its detection (18–21) At present, a large amount of research has indicated that STAT6 is the most sensitive and specific marker for the diagnosis of SFTC (13), and the 96% positive rate of STAT6 in this study is consistent with this. At the same time, we found that the positive rate of Vimentin is 100%. Therefore, a combination of these above-mentioned markers can be used for the diagnosis of SFTC.

These methods can also be used to distinguish malignant SFTC from benign SFTC. Cardillo et al. (8) identified risk factors for the malignancy of SFTP, which are uneven density, unclear boundaries, thickened surrounding pleura, and excessive length (>10 cm). Song et al. (22) reported that cystic change, necrosis, and bleeding in tumors and excessive length (>10 cm) are common phenomenon of malignant SFTC. In this study, although we did not find that tumor size and location have a diagnostic value for malignant SFT, in the CT findings necrosis in the lesion indicated malignancy. In addition, there was no difference in immunohistochemical results between benign and malignant SFTP, except with respect to the Ki-67 index. Therefore, CT scanning, especially using high-resolution multi-slice spiral enhanced CT, can be a reliable method for distinguishing malignant from benign SFTC. The pathological criteria for SFTC identified previously are as follows: 1) increased cell density and active cell growth; 2) pleomorphism; 3) mitotic figures ≥4/10 HPF; and 4) neoplastic necrosis and/or infiltrating edges. In this study, we found that malignancy is correlated with frequent occurrence of cell nuclear division, significant morphological variation, necrosis, and a high Ki-67 index, but not with tumor size or location.

Surgery is still the main method used for SFT treatment, and the particular surgical approach employed is mainly based on the size of the tumor, as follows: 1) video-assisted thoracic surgery (VATS) is often used for visceral pleura tumors attached by a pedicle with a diameter of <4 cm; 2) auxiliary small incision thoracotomy is widely used for wide-based tumors 5–8 cm in size; and 3) conventional thoracotomy is utilized when the tumor is >10 cm. However, we used VATS for three patients with tumors >10 cm, and all of them obtained an excellent prognosis. Furthermore, in the postoperative follow-up to the end of the study, no metastasis or recurrence was found. It is worth mentioning that complete resection is an important factor affecting local recurrence and metastasis (23), and we concluded that complete resection of the tumor is an effective method for treating SFT. In addition to surgery, radiofrequency ablation and photodynamic therapy can be used for unresectable SFT. In this study, two patients received radiofrequency ablation, which yielded a good prognosis. Previous studies have shown that patients with SFT tend to exhibit a terrible prognosis (24). However, in this study, we found that nearly all the patients had an excellent prognosis. Overall, we found that surgery, radiofrequency ablation, and photodynamic therapy are reliable methods for treating SFT.

In this study, we analyzed the diagnosis and treatment of SFT, but there are some limitations to our research. Firstly, the number of cases we investigated was small, and we did not compare the effectiveness and safety of open surgery and laparoscopic surgery. In our study, due to the small sample size at present, and the vast majority of patients received traditional surgical treatment, postoperative adjuvant chemotherapy and other systemic treatment were not carried out, and the number of patients with new treatment methods such as radiofrequency ablation and hyperthermia was less, so the comparison between traditional surgical treatment and new treatment was not carried out for the time being. Our future studies will address these two issues.

In sum, our analysis of diagnostic methods and treatments for SFT concluded that middle age, chest tightness, necrotic CT manifestations, and a high Ki-67 index are more common in malignant SFTP, and these findings can provide direction for clinicians.

## Data Availability Statement

The original contributions presented in the study are included in the article/**Supplementary Material**, further inquiries can be directed to the corresponding author.

## Ethics Statement

The studies involving human participants were reviewed and approved by the Henan Institute of Respiratory Diseases, The First Affiliated Hospital of Zhengzhou University ethics committee. The patients/participants provided their written informed consent to participate in this study. Written informed consent was obtained from the individual(s) for the publication of any potentially identifiable images or data included in this article.

## Author Contributions

ZJ conceived the idea and conceptualized the study. LJ collected the data. ZZ analyzed the data. ZJ drafted the manuscript, then TB reviewed the manuscript. All authors contributed to the article and approved the submitted version.

## Conflict of Interest

The authors declare that the research was conducted in the absence of any commercial or financial relationships that could be construed as a potential conflict of interest.
