# Erratum: Solitary Fibrous Tumors of The Chest: An Analysis of Fifty Patients

**DOI:** 10.3389/fonc.2021.741181

**Published:** 2021-07-27

**Authors:** Frontiers Production Office

**Affiliations:** Frontiers Media SA, Lausanne, Switzerland

**Keywords:** solitary fibrous tumor of the chest, characteristic, diagnosis, treatment, retrospective analysis

Due to an editorial error, the article was published as a Review article. The correct article type is Original Research. The publisher apologizes for this mistake.

The original version of this article has been updated.

