# Supplementary material for: Solitary Fibrous Tumors of the Chest: An Analysis of Fifty Patients
Source: Front Oncol. 2021 Jul 1;11:697156. doi: 10.3389/fonc.2021.697156 (PMC8280784; doi:10.3389/fonc.2021.697156)
Supplement: Supplementary file 1 [file Table_1.doc]

**Supplement Table 1. Detailed baseline [characteristic](https://fanyi.so.com/?src=onebox#characteristic)s of patients**

| Number | Age | Gender | Smoking | Cough | Expectoration | Chest tightness | Fever | Chest pain | Location | Nature of tumor | Follow up |
| --- | --- | --- | --- | --- | --- | --- | --- | --- | --- | --- | --- |
| 1 | 57 | Male | No | With | With | Without | Without | Without | Left mediastinum | Begign | Alive |
| 2 | 58 | Female | No | Without | Without | Without | Without | Without | Right chest cavity | Begign | Alive |
| 3 | 42 | Female | No | Without | Without | Without | Without | Without | Right chest cavity | Begign | Alive |
| 4 | 49 | Female | No | Without | Without | Without | Without | Without | Left chest cavity | Begign | Alive |
| 5 | 28 | Female | No | With | With | Without | Without | With | Left chest cavity | Begign | Lost |
| 6 | 53 | Female | No | Without | Without | Without | Without | Without | Right chest cavity | Begign | Alive |
| 7 | 51 | Male | Yes | With | With | With | Without | Without | Left chest cavity | Begign | Alive |
| 8 | 50 | Female | No | With | With | Without | Without | Without | Left chest cavity | Begign | Alive |
| 9 | 57 | Female | No | Without | Without | Without | Without | Without | Right chest cavity | Begign | Alive |
| 10 | 44 | Male | No | Without | Without | Without | Without | Without | Left chest cavity | Begign | Alive |
| 11 | 53 | Female | No | Without | Without | Without | Without | Without | Right chest cavity | Begign | Alive |
| 12 | 77 | Male | Yes | Without | Without | Without | Without | Without | Right mediastinum | Begign | Alive |
| 13 | 47 | Male | Yes | Without | Without | Without | Without | Without | Right mediastinum | Begign | Alive |
| 14 | 43 | Male | No | Without | Without | Without | Without | Without | Right chest cavity | Begign | Lost |
| 15 | 53 | Female | No | With | With | With | Without | Without | Left chest cavity | Begign | Lost |
| 16 | 47 | Female | No | Without | Without | Without | Without | Without | Right chest cavity | Begign | Alive |
| 17 | 47 | Female | No | With | With | With | Without | With | Left chest cavity | Begign | Alive |
| 18 | 51 | Female | No | Without | Without | Without | Without | With | Left chest cavity | Begign | Alive |
| 19 | 68 | Male | Yes | Without | Without | Without | Without | Without | Left mediastinum | Begign | Alive |
| 20 | 51 | Female | No | With | Without | With | Without | With | Left chest cavity | Begign | Alive |
| 21 | 84 | Male | No | Without | Without | Without | Without | Without | Left chest cavity | Begign | Dead |
| 22 | 59 | Female | No | Without | Without | Without | Without | With | Left chest cavity | Begign | Alive |
| 23 | 47 | Female | No | With | Without | Without | Without | Without | Right chest cavity | Begign | Lost |
| 24 | 54 | Male | Yes | Without | Without | Without | Without | Without | Right chest cavity | Begign | Alive |
| 25 | 46 | Female | No | Without | Without | Without | Without | Without | Right chest cavity | Begign | Alive |
| 26 | 60 | Female | No | With | Without | With | Without | Without | Right chest cavity | Malignant | Alive |
| 27 | 44 | Male | No | With | Without | Without | Without | Without | Left mediastinum | Malignant | Alive |
| 28 | 61 | Male | No | Without | Without | Without | Without | Without | Left chest cavity | Malignant | Alive |
| 29 | 59 | Female | No | With | With | With | Without | With | Left mediastinum | Malignant | Alive |
| 30 | 80 | Female | No | Without | Without | With | Without | Without | Left chest cavity | Malignant | Dead |
| 31 | 56 | Male | No | Without | Without | With | Without | Without | Right chest cavity | Malignant | Alive |
| 32 | 60 | Male | Yes | With | With | With | Without | Without | Left chest cavity | Malignant | Alive |
| 33 | 52 | Female | No | Without | Without | Without | Without | Without | Right chest cavity | Malignant | Alive |
| 34 | 57 | Female | No | With | With | Without | Without | Without | Right lung | Begign | Alive |
| 35 | 60 | Male | No | Without | Without | Without | Without | Without | Right lung | Begign | Alive |
| 36 | 42 | Male | Yes | With | Without | Without | Without | Without | Right lung | Begign | Alive |
| 37 | 39 | Male | No | With | With | Without | Without | Without | Right lung | Begign | Alive |
| 38 | 67 | Male | No | With | With | Without | Without | Without | Right lung | Begign | Alive |
| 39 | 62 | Female | No | Without | Without | Without | Without | Without | Right lung | Begign | Lost |
| 40 | 42 | Female | No | Without | Without | Without | Without | With | Right lung | Begign | Alive |
| 41 | 47 | Male | No | With | With | With | With | With | Right lung | Begign | Alive |
| 42 | 55 | Female | No | With | Without | Without | Without | Without | Left lung | Begign | Lost |
| 43 | 52 | Male | Yes | Without | Without | Without | Without | With | Left lung | Begign | Alive |
| 44 | 53 | Female | No | Without | Without | With | Without | Without | Left lung | Begign | Alive |
| 45 | 48 | Female | No | Without | Without | Without | Without | Without | Left lung | Begign | Lost |
| 46 | 46 | Female | No | Without | Without | Without | Without | Without | Left lung | Begign | Lost |
| 47 | 45 | Male | Yes | Without | Without | Without | Without | Without | Left lung | Begign | Alive |
| 48 | 62 | Female | No | Without | Without | Without | Without | Without | Left lung | Begign | Alive |
| 49 | 64 | Male | Yes | With | With | Without | Without | With | Left lung | Begign | Lost |
| 50 | 55 | Female | No | Without | Without | Without | Without | Without | Left lung | Begign | Alive |
